# Long-term monitoring of coral reef fish assemblages in the Western central pacific

**DOI:** 10.1038/sdata.2017.176

**Published:** 2017-12-05

**Authors:** Adel Heenan, Ivor D. Williams, Tomoko Acoba, Annette DesRochers, Randall K. Kosaki, Troy Kanemura, Marc O. Nadon, Russell E. Brainard

**Affiliations:** 1 Joint Institute for Marine and Atmospheric Research, University of Hawaii, Manoa, Honolulu, Hawaii 96822, USA; 2 NOAA Pacific Islands Fisheries Science Center, Honolulu, Hawaii 96818, USA; 3 Papahānaumokuākea Marine National Monument, NOAA, Honolulu, Hawaii 96818, USA

**Keywords:** Biogeography, Ecosystem ecology, Marine biology

## Abstract

Throughout the tropics, coral reef ecosystems, which are critically important to people, have been greatly altered by humans. Differentiating human impacts from natural drivers of ecosystem state is essential to effective management. Here we present a dataset from a large-scale monitoring program that surveys coral reef fish assemblages and habitats encompassing the bulk of the US-affiliated tropical Pacific, and spanning wide gradients in both natural drivers and human impact. Currently, this includes >5,500 surveys from 39 islands and atolls in Hawaii (including the main and Northwestern Hawaiian Islands) and affiliated geo-political regions of American Samoa, the Commonwealth of the Northern Mariana Islands, Guam, and the Pacific Remote Islands Areas. The dataset spans 2010–2017, during which time, each region was visited at least every three years, and ~500–1,000 surveys performed annually. This standardised dataset is a powerful resource that can be used to understand how human, environmental and oceanographic conditions influence coral reef fish community structure and function, providing a basis for research to support effective management outcomes.

## Background & Summary

Coral reefs ecosystems are critically important to people; they provide food and livelihoods to millions of people worldwide^1^ and contribute to the cultural fabric of coastal communities. They are also important from a global heritage perspective, due to the intrinsic value of the biodiversity and richness of life they contain^2^. Globally, we have just experienced the third and longest mass coral reef bleaching event on record^3^. The dramatic loss of living coral and reef-associated populations caused by human-induced climate change^4,5^ threatens the integrity of coral reef ecosystems worldwide. Around human population centres, the impacts of climate change are typically compounded by multiple other stressors, such as unsustainable fishing practices and land-based sources of pollution^6,7^. There is therefore an urgent need to better understand the natural geographic and environmental variability of these systems, along with the key drivers of change, to inform and promote effective coral reef ecosystem management.

Large-scale and long-term monitoring datasets have an important role to play in this process. In particular, the implementation of standardised monitoring methods across gradients of oceanographic conditions and levels of human impact yields a powerful data resource that can be used to better understand the natural variability and differential susceptibility of coral reef ecosystems to local and global drivers. Here, we present such a dataset, which is collected for a long-term monitoring program, the National Oceanic and Atmospheric Administration (NOAA) Pacific Reef Assessment and Monitoring Program (RAMP). The NOAA Ecosystem Science Division (ESD) and partners have implemented RAMP—i.e., multi-disciplinary coral reef ecosystem monitoring—across U.S. and U.S.-affiliated territories in the Western and Central Pacific Ocean since 2000. The focus of this data descriptor is the reef fish and paired benthic habitat-monitoring component of Pacific RAMP that has been implemented since 2010, that being the entire period in which the survey design and monitoring methods have followed those specified in the National Coral Reef Monitoring Plan (NCRMP)^8^.

Prior to 2010, the Pacific RAMP used different survey methods and statistical sampling design to assess fish populations. Specifically, each region was visited every 2 years, with reef fish surveys conducted using belt-transects at haphazardly-located sites. Following a 2-year methods comparison period between 2007 and 2009, the reef fish-monitoring component was revamped into its current form, with the aims of systematising the design, maximizing survey site replication, and broadening the survey domain. To a large degree, the current Pacific RAMP statistical sampling design and survey methods were modelled on the fishery-independent diver visual survey program conducted in Florida by the NOAA Southeast Fisheries Science Center and partners^9^. The overarching goal that motivated the change was to generate data representative of coral reef hard-bottom substrate at the islands-scale for the numerous Pacific jurisdictions covered by our program. To that end, a wide (39 islands surveyed) but thin (~3 days per island per survey cycle, approximately 30–50 sites per island) was adopted. Around that time, we also shifted from a 2-year cycle to a 3-year cycle (i.e., each jurisdiction surveyed once every three years). Pacific RAMP surveys have been supplemented by additional survey efforts around American Samoa, Hawaiʻi and Guam and by data gathered on monitoring cruises led by Papahānaumokuākea Marine National Monument (PMNM)—in all cases using identical methods, design and, often, the same personnel.

This data descriptor is limited to data collected from 2010 onwards using a stationary point count (SPC) survey method and a randomized depth-stratified design. The survey domain for Pacific RAMP is all hard-bottom substrate in ≤30 m depth. In addition to fish counts, divers visually estimate benthic cover and habitat structural complexity, so that each fish count is paired with habitat information.

Between 2010 and 2017, ~4,700 such surveys were conducted across 39 islands and atolls. The data collected serve four main purposes: 1) to fulfil NCRMP mandates to assess the status and trends of reef fish assemblages across coral reefs of the U.S.^8^; 2) to provide data suitable to assess the status of coral reef fisheries stocks^10,11^; 3) to support federal and jurisdictional management by providing a broad spatial context to status and trends apparent from, generally, spatially smaller-scale surveys conducted by those agencies^12^; and 4) to generate a consistent and large-scale dataset as a resource for the scientific community^13–18^.

## Methods

**Figure 1:**
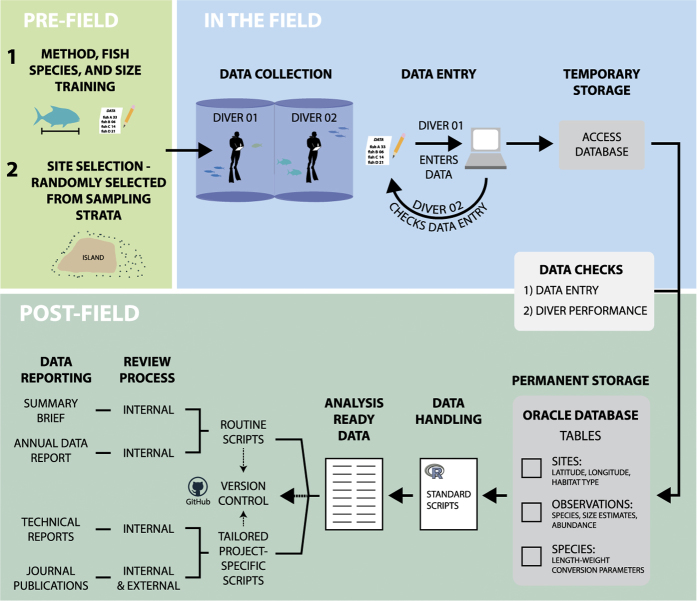
The pre-field, field and post-field components of monitoring data collection. For the dataset published here, this includes the training, data collection and entry as well as data processing and reporting steps.

The data cycle spans three different steps: pre-field, in the field and post-field (Fig. 1). Prior to field data collection, we selected sites via a randomized depth-stratified design. In the field, the monitoring team accessed the survey regions on board the NOAA Ships *Hi*ʻ*ialakai* and *Oscar Elton Sette*, but daily work was conducted from small boats that were launched from the ship and recovered each day. Survey divers collected data while using open-circuit SCUBA and entered data into a relational database (Microsoft Access). Upon completion of a survey mission, the data were migrated to an enterprise relational database (Oracle), and subsequently synthesized, and processed into analysis-ready data using standard scripts. Each of the three steps are described in turn below, and more detail is available in our standard operating procedure document^19^.

### Statistical sampling design

Monitoring occurred in four regions; American Samoa, the Mariana Archipelago (the Commonwealth of the Northern Mariana Islands and Guam), Hawaiʻi (the main and Northwestern Hawaiian Islands) and the Pacific Remote Islands Areas (an administrative rather than geographic grouping). The goal was to survey reefs as widely as possible—i.e., survey effort is spread across many islands and atolls and, within each reef area, across as wide a domain as feasible—in this case all hard-bottom substrate in water shallower than 30 m. In total, 39 islands and atolls spread across the U.S. Pacific territories are surveyed for this program (Table 1). The 30-m depth limit is a safe-diving regulation. Typically, 3–5 days were spent at each island or atoll during each visit (generally once every 3 years), conducting 30–50 fish surveys during that time. Each island or atoll (henceforth ‘reef area’) is stratified by reef zone (backreef, forereef, protected slope, or lagoon, although the majority of reef areas only have forereef) and by depth zone: shallow (0–6 m), mid (6–18 m), and deep (18–30 m). In addition, there is a level of stratification based on ‘sector’ (i.e., section of coastline and/or management status). Sectors are only utilized at a number of the larger populated islands where there can be very different levels of management (e.g., protected areas), human population density or access. For example, Guam is subdivided into three sectors: ‘Marine Preserve’ (being all areas within Guam’s Marine Preserve System); ‘Guam Open East’ (areas outside of Marine Preserves on east side of Guam); and ‘Guam West.’ Similarly, the main Hawaiian Islands and Tutuila have between two and seven sectors per island, with sector boundaries designed to reflect broad differences in oceanographic exposure, reef structure, local human population density and management status. At the majority of the inhabited islands, supplemental survey operations or additional survey days during routine cruises, have allowed for higher sampling density around those human population centres than at remote islands. Finally, three neighbouring islands in the northern Commonwealth of the Northern Mariana Islands (CNMI)—Alamagan, Guguan and Sarigan (‘AGS’)—are routinely pooled into a single statistical sampling and reporting unit, as their small size makes it infeasible to allocate sufficient time (and therefore number of surveys) for us to be able to generate meaningful summary metrics for each island individually. The statistical sampling design, terminology and reporting units are summarized in Table 2. A summary of the number of sites surveyed per island per habitat strata is presented in Table 3.

### Site selection

Prior to each survey mission, sample site locations were randomly selected from geographic information system (GIS) substrate and strata maps maintained by the ESD. These maps were created using information from the NOAA National Centers for Coastal Ocean Science (NCCOS), reef zones (e.g., forereef) and geomorphologic structures digitized from IKONOS satellite imagery or nautical charts, bathymetric data from the ESD-affiliated Pacific Islands Benthic Habitat Mapping Center, University of Hawaiʻi at Mānoa, and prior knowledge gained from previous visits to survey locations.

Logistical and weather conditions factor into the planning and allocation of survey effort around each island. Small islands can be assumed to be randomly surveyed in their entirety i.e., all stretches of coastline can have a random site assigned to it, as these islands, weather permitting, can typically be circumnavigated by a small boat in a day. For islands too large to sample in their entirety, we break the coastline into 4–6 fixed evenly spread out sections, in which random sites are assigned to. Prior to data collection, these constraints determined the section of target habitat from which sites are randomly selected and the position of the ship during the survey mission. Prior to each cruise, the target number of sites per stratum is determined by proportionally allocating total expected sites at the reef areas (generally 30–50) based on a weighting factor calculated from the size of the strata and the variance of the target output metrics (e.g., consumer group biomass and total fish biomass), and adjusted for what is feasible given operational constraints—e.g., the safety limit on frequency and duration of deep dives.

### Survey method

At each reef fish survey site, two types of data are collected as part of a rapid ecological assessment (REA): visual counts of the fish assemblage and an assessment of benthic habitat including site characteristics such as water clarity and depth. We use a form of stationary point count (SPC)^19^, which involves a pair of divers conducting simultaneous counts in adjacent, visually estimated 15-m diameter cylindrical plots extending from the substrate to the limits of vertical visibility. Prior to beginning each SPC pair, a 30-m gray polyester transect line is laid across the substratum. Markings at 7.5, 15 and 22.5 m enable survey divers to locate the midpoint (7.5 m or 22.5 m) and two edges of their survey plots. Prior to 2015, divers visually estimated water visibility, but since 2015 horizontal visibility on survey sites has been measured using a Secchi disc. To do this, the diver who laid out the transect swims back down the 30 m transect line towards the second diver who, positioned at 0 m, holds a slate up with black and white Secchi quadrants. The first diver then estimates water clarity as the point along the line where the black and white Secchi disc quadrants become visible.

### Surveying the reef fish assemblage.

Each fish count consists of two main parts. The first of these is a 5-min species enumeration period in which each diver generates a list of the taxa observed within their cylinder—to species if possible. Divers record the taxa using four-letter codes, which are linked to a species table in the database with full names and other information about each species. Species identification is based on the assessment of experienced fish survey divers, who are trained using the Fish SPC Method training package available on the Pacific Islands Fisheries Science Center webpage (https://www.pifsc.noaa.gov/cred/survey_methods/fish_surveys/rapid_ecological_assessment_of_fish-survey_method_training.php), and who verify species identification with various sources (e.g., www.fishbase.org^20–22^). The species names and biogeographic distribution are based on the World Register of Marine Species (WoRMS)^23^. At the end of the 5-min period, divers begin the tallying portion of the count, during which they systematically work through their species list, recording the number and estimated size (total length, TL, to the nearest cm) of fishes present within the cylinder. The tallying portion is conducted as a series of rapid visual sweeps of the plot, with one species-group (e.g., mid-water surgeonfish, benthic butterflyfish) counted per sweep. To the extent possible, divers remain at the centre of their cylinders throughout the count. However, small, generally site-attached and semi-cryptic species, which tend to be undercounted by a diver remaining in the centre of a 15-m diameter cylindrical plot, are left to the end of the tally period, at which time the diver swims through the plot area carefully searching for those species. In cases where a species is observed during the enumeration period but was not present in the cylinder during the tallying period, divers record their best estimates of size and number from their observations in the first encounter during the enumeration period and mark the data record as ‘non-instantaneous.’ Since 2012, we have recorded three additional types of observations: 1) when a species was first observed in the cylinder between 5 and 10 min into the survey (i.e., in the first 5 min of the tallying portion), the diver conducts a rapid visual sweep of their cylinder for that species and records the number and size as ‘five-to-ten’; 2) data on species first observed inside the cylinder any time after that, up to 30 min into the survey, are recorded as ‘ten-to-thirty’; and 3) the presence of other species of interest in the general vicinity of the survey cylinder, and seen at any time throughout the survey period are recorded as ‘present’. In cases where divers first saw cryptic or previously unobserved fishes mid-way through a survey, they use their discretion to decide whether to record them as instantaneous or another category. For example, a camouflaged scorpionfish within the survey area but not observed until late in the count was likely there throughout the entire period of the count and could reasonably be recorded as an ‘instantaneous’ count. Surveys are not conducted if horizontal visibility is <7.5 m (i.e., when divers cannot distinguish the edges of their cylinder).

### Surveying the reef habitat.

After completing the fish survey, both divers scan the benthos in their survey cylinder for 2–3 min and visually estimate the percentage cover of: encrusting algae, fleshy macroalgae, hard coral, sand and other (turf algae, soft coral and cyanobacteria grouped together). Divers also record the depth at the centre and high- and low-edges of their cylinders (the latter two values providing a measure of slope), broad habitat type and structural complexity. Since 2012, divers record reef habitat complexity by visually estimating the percentage of the cylinder that falls into a series of bins representing different heights from the plane of the reef: <0.20 m, 0.20–0.50 m, 0.50–1 m, 1–1.5 m and >1.5 m. Prior to 2011, divers estimated reef substrate complexity on a five point scale (1–5). Divers also record the maximum height of substrate within their cylinders. Diver conduct a rapid visual assessment on the abundance of ‘free’ (e.g., *Tripneustes* spp., *Heterocentrotus* spp., *Diadema* spp. and *Echinothrix* spp.) and ‘boring’ (e.g., *Echinometra* spp. and *Echinostrephus* spp.) urchins using a DACOR scale each urchin category (for free urchins those are: D: Dominant [>100], A: Abundant [51–100], C: Common [21–50], O: Occasional [6–20], R: Rare [<5]; for boring urchins categories are D: Dominant [>500], A: Abundant [251–500], C: Common [101–250], O: Occasional [26–100], R: Rare [<25]). Finally divers identify the broad-scale habitat type in the general area of the survey. The habitat codes follow the geomorphological structures identified by the NCCOS^24^: 1) aggregate reef; 2) aggregated patch reefs; 3) aggregated patch reef (i.e., an individual patch reef); 4) pavement; 5) pavement with patch reefs; 6) pavement with sand channels; 7) rock/boulder; 8) reef rubble; 9) spur and groove; and 10) sand with soft coral or rock.

### Data processing

#### Calculating fish biomass and benthic cover estimates per site

Using the count and size estimate data collected per diver in each replicate cylinder survey, the body weight of individual fish is calculated using length-to-weight (LW) conversion parameters, and, where necessary, length-length (LL) parameters [e.g., to convert TL to fork length (FL) for species with LW parameters based on FL]. LW and LL conversion parameters are largely taken from two sources^25,26^.

$$W={\mathrm{aL}}^{b}$$

Where W is weight in grams, L is length in centimeters and a and b are constants. The term ‘biomass’ herein refers to the aggregate body weight of a group of fishes per unit area (g m^−2^). The diver-level data that are collected in adjacent cylinders at the same survey site are not independent replicates; therefore, a survey is always the combined data from the adjacent cylinders and this combined site-level data is the base sample unit of survey data. Site-level estimates (e.g., abundance, biomass, benthic cover, complexity) are calculated by taking the mean of the values from the adjacent diver-level counts conducted for each survey. Site-level fish metrics (e.g., abundance and biomass) can be pooled into the standard Pacific RAMP consumer group classification. The consumer groups are Primary Consumer, Secondary Consumer, Planktivore and Piscivore, and are based largely on diet data from FishBase.

#### Code availability

The code to produce site-level summary metrics from the observation level data records are provided in Supplementary Files 1 and 2.

## Data Records

The complete Pacific RAMP fish SPC dataset (2010–2017) (Data Citation 1: xxx) is provided as a comma-separated file named NOAA_PACIFIC_RAMP_FISH_SPC_2010_2017_SCI_DATA_.csv. Each data record in the data file is an estimated count and size of a single fish taxa from a single observer (DIVER) at a single cylinder as part of a single survey at a particular site. Each record includes an observation type (see method description) along with metadata that relates to the species observed. Each data record has a unique numeric identifier (SITEVISITID) that relates to the survey (i.e., one dive at one site by one group of divers). Each site, i.e., survey location, also has a unique identifier (SITE) and because we do not revisit survey locations, SITE is also a unique identifier for the survey. Typically a site contains data records from a single SPC-pair, i.e., the two adjacent cylinders that are simultaneously surveyed by the dive team. Meta-data on benthic habitat, site location and sampling date relate to all data records within the same survey. Each column field in the provided data file is explained in full in Table 4 (available online only).

The processing code to generate a variety of survey-level summary metrics from the data records in the data file is provided as Supplementary File 1, this code depends on loading the custom set of functions provided as Supplementary File 2. The dataset we are providing is derived from the raw observation level data we store in Oracle at the Pacific Islands Fisheries Science Center. It differs in that redundant fields from the base data export from Oracle are excluded, diver identifiers are converted to unique numeric codes to preserve their anonymity and obsolete benthic habitat categories are removed.

## Technical Validation

Underwater visual censuses (UVCs) are commonly used to survey fish assemblages and benthic habitats for coral reef ecosystems. Potential sources of error within UVC methods include: inter- and intra-diver variability; the depth and time restrictions associated with using SCUBA, which limit more detailed assessments; differential detectability of species due to the habitat and environment (i.e., highly complex versus less complex habitats) or the behavioural interaction (attraction or repulsion) between divers and fish species. Estimates of fish abundance can also vary depending on the UVC method of choice. The impact that these potential sources of uncertainty can have on the quality of these data presented here are discussed in turn.

**Figure 2:**
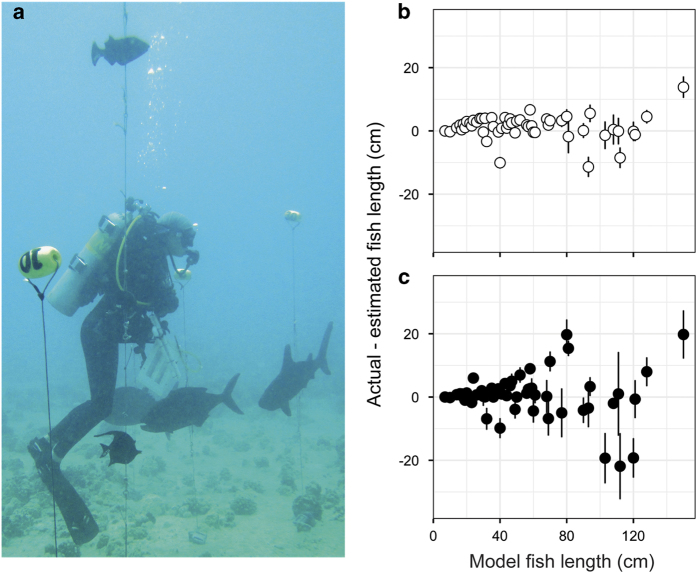
Fish size estimation training trials by experienced and trainee survey divers. During training dives, observer accuracy is assessed by divers estimating the size of wooden fish models of known lengths (**a**), which are haphazardly distributed throughout a mock SPC cylinder. Example mean difference (+—standard error) between actual and estimated length of model fishes by trained staff (**b**—open circles) and by trainee survey divers (**c**—closed circles) during size estimation training trials between 2014–2016. The closer the difference between size estimates and actual model sizes is to zero, the more accurate the sizing. Trainee fish survey divers, which includes novices in the middle of the training program, people who have done fish surveys but not the SPC method, as well as people who are taking an SPC refresher tend to towards more variable size estimates compared to core staff. Typically new fish surveyors are required to have conducted a minimum of 30 survey dives prior to joining a RAMP cruise.

We address intra- and inter-diver variability in this dataset in two ways, both of which are considered a part of our routine data quality and validation procedures. Firstly, new divers who collect fish and benthic data for Pacific RAMP are trained in both fish identification and the survey protocol, in classroom and in-water sessions. The complete training package for CREP fish divers is available at: https://www.pifsc.noaa.gov/cred/survey_methods/fish_surveys/rapid_ecological_assessment_of_fish-survey_method_training.php. Prior to each cruise, all divers (whether new or experienced) must accurately identify >90% of fishes shown in a regional-specific fish species identification test. This test is intended to be difficult—in that it is weighted towards rare species and those that have conspecifics with similar appearance. Secondly, outside of the survey cruise season, fish divers take part in regular training exercises, typically on a bimonthly basis. Generally, this in-water training includes two dives: one to conduct a practice SPC survey, including the benthic habitat assessment, and another to estimate fish sizes, using a series of fish models of various sizes from 10 to >150 cm. Divers’ size estimates are then compared against the known sizes of the fish models used in that test (Fig. 2).

The remainder of our routine data quality and checking methods occur in the field, where we typically have between 4 and 10 fish survey divers. The dive buddy pairs are regularly mixed up throughout a survey cruise, and divers routinely discuss and compare species identification and sizes in the field. This is done immediately after a survey, as well as during the data entry stage, when divers check data entered by their diver partner against their datasheet for sizing, species identification and data entry errors. The difference between the estimates of each diver and those of their dive partner at each site is calculated, and referred to as diver performance. This can be done for any parameter estimated, but during field operations, we assess total fish biomass, species richness (number of unique species counted), the size distributions of commonly observed species, and benthic cover. Real differences between dive partners are expected, as divers survey adjacent cylinders, but not identical areas of reef. However, if there is no consistent bias in the estimates made by a diver, the median difference with their partner should be close to zero (i.e., half their estimates being higher than their partners’ half lower). Boxplots of diver performance, therefore, give 1) a strong but general indication of relative bias; if there is not consistent bias, then the median differences between a single diver and their dive partners will be close to zero, and 2) an indication of how variable each divers’ counts are compared to their dive partners. We generate boxplots of diver performance every few days to provide feedback on diver performance relative to the rest of the team and to allow for the early detection of observer error^27^.

Experienced survey divers are capable of accurately estimating coral cover based on visual assessments of the survey area^28,29^. During the fish surveys both a rapid visual assessment and a photo-quadrat survey of the benthos (not part of this Data Descriptor) are conducted after the fish count. From an earlier study comparing the visual assessment with photo-quadrats at the Pacific RAMP SPC survey sites, we know that, relative to photo-quadrat surveys of the same survey plot, divers tend to underestimate hard coral cover (by −3%), and encrusting algae (−2.3%) and overestimate fleshy macroalgae (6.5%)^30^. Rapid visual estimates have greater scope for observer-bias relative to the photo-transect method. Nevertheless, we believe this method provides a coarse but meaningful and immediately available estimate of benthic cover at the functional group level, estimates that are suitable for characterizing the benthos at a survey-site.

As with all visual survey techniques of fish assemblages, survey counts are affected by imperfect detectability. With the SPC this is particularly true for very small, cryptic and nocturnally active species. We consider the detectability of fishes in a SPC survey similar to other common whole assemblage reef fish survey techniques (such as a belt transect), because: 1) divers remain within the same survey area throughout the survey, therefore have multiple opportunities to observe species present within their cylinders; 2) fishes are counted in a series of rapid sweeps of the cylinder with similar species grouped together (i.e., divers are focused on one search image per sweep); this prevents divers form being overwhelmed by the exceptional abundance and high diversity we encounter at some—particularly remote- locations we survey and; 3) divers carefully swim through their cylinder at the end of the survey, recording species and fishes that may have been missed. Methods that survey larger areas of reef, such as a long belt transect, may allow for greater detectability of skittish fishes that move relatively far ahead of the survey divers, before they move out of the survey area. However, as we record observations of species for up to 30 min after the start of the survey, the SPC method provides opportunities to record fishes that return to the survey area, if divers are not perceived as a threat^31^.

Differences in fish behaviour in response to diver presence can be a source of bias in survey estimates. For example, target fishes may be wary of divers in locations where they have come to associate divers with a risk of being hunted; and alternatively, curious fishes may be more likely to approach divers closely enough to be counted in remote locations where divers are not perceived as a threat^32^. Although that effect can be substantial in some cases^33^, the scale of those effects varies among locations and depending on method used. In an attempt to quantify one important component of the disturbance caused by divers, we recently compared counts by divers using our methods on SCUBA (which emit noisy and conspicuous bubbles) with counts made by divers using closed circuit re-breathers (CCR, which do not) at a range of locations in the main Hawaiian Islands. While there were significantly higher counts of target fishes by CCR divers around the most heavily fished location (Oʻahu), those effects were much smaller than those reported around heavily fished parts of Guam^33^, and we found no clear effect at other locations, including Maui-nui where there is still considerable fishing effort^34^. Diver avoidance is clearly a potential concern for all underwater survey programs, but the evidence available indicates that these effects are relatively insignificant except at the extreme high-end of fishing pressure.

Each underwater visual survey method has its strengths, weaknesses and inherent biases. One of the more common UVC methods is the belt transect, and indeed before 2010, when we fully adopted the current survey design using the SPC method, our program monitored fishes via multiple 25-m belt transects per site. Between 2007 and 2009, we co-located belt and SPC surveys at 332 sites, across the regions surveyed for Pacific RAMP. Comparisons of the data generated by these two different methods indicated that (i) densities were similar for most taxa; (ii) SPC data tended to have lower variability (with the exception of small, benthic associated fishes such as damselfish, wrasse and hawkfish); and that (iii) the SPC method tended to generate fewer zero counts for most taxa than belt surveys (Supplementary File 3). Clearly these results are highly dependent on the specific variation of the belt method we implemented, but this comparison justified our program’s adoption of the SPC approach from 2010 on.

One aspect of the SPC that makes it suitable for the purposes of Pacific RAMP is our recording of ‘instantaneous’ count data, which are equivalent to a series of snapshots of fish presence. Divers systematically record one group of fishes at a time, carefully estimating their sizes in one sweep, rather than requiring divers to count and size multiple groups simultaneously. This instantaneous data is used to generate density estimates per unit area. In contrast, methods such as the belt transect typically record fishes present in, moving into or across a survey area ahead of the diver during some -often undefined- period of time. Depending on the purpose of the survey, it can be beneficial that open counts provide increased opportunities to record observations of mobile species, but time integrated counts of ‘open’ survey areas, like the belt transect will tend to overestimate the density of mobile species—potentially substantially^35^.

Indeed, the biomass estimates derived from the Pacific RAMP surveys tend to be lower than those from other reef fish surveys in the Pacific^36^. This is likely, in part, due to the aforementioned methodological differences between the SPC and the more commonly implemented belt transect method. The lower biomass recorded in this dataset could be due to our sampling design. In particular, the sampling domain of Pacific RAMP is likely broader than most other reef fish surveys. Specifically, we sample all hard-bottomed habitats in less than 30-m, which includes considerable areas of low relief and low coral cover habitats that typically have lower biomass than more structurally-complex habitats that are the focus of most survey programs (e.g., spur and groove or aggregate reef). Survey method choice and statistical sampling design can have large impacts on reef fish biomass estimates produced by reef fish surveys and, therefore, we caution data users against simply blending the data provided here together with data from other sources and recommend that the biomass estimates generated should always be considered as a relative, rather than an absolute, measure when compared to other data sources.

## Usage Notes

The code to generate site-level estimates of summary fish and benthic estimates from the raw observations is available with this paper (Supplementary Files 1 and 2). Pooling these data to generate island-level estimates requires knowledge of the statistical sampling scheme for each year and whether there were any additional projects that deviate from the standard Pacific RAMP design, such as an intensive survey effort within a particular bay. For this reason, we encourage data users to contact us (email: nmfs.pic.credinfo@noaa.gov with subject line: For the Attention of the Fish Team Lead) to discuss how best to handle these instances.

Users of these Pacific RAMP reef fish and paired benthic survey data should be aware of the following aspects of the dataset:

1. The different observation types. Data are recorded as one of five different ‘observation types.’ The majority of records—those where a species is observed during the enumeration period and where individuals of that species are present in the cylinder at the time of the tallying portion for that taxa—are recorded as ‘instantaneous’ observations (OBS_TYPE=‘I’). When a species is observed during the enumeration period but is not present during the instantaneous sweep for that taxa, divers record size and number present in the cylinder when it was first observed during the enumeration period and mark the data record as ‘non-instantaneous’ (OBS_TYPE=‘N’). Since 2012, we also record three other types of observations: 1) when a species is first observed in the cylinder between 5 and 10 min into the survey (i.e., in the first 5 min of the tallying portion), the diver conducts a rapid visual sweep of their cylinder for that species and records number and size as ‘five-to-ten’ (OBS_TYPE=‘F’); 2) when a species is first observed inside the cylinder any time after that, up to 30 min into the survey, the diver records the number and size as ‘ten-to-thirty’ (OBS_TYPE=‘T’); and 3) when the presence of other species of interest in the general vicinity of the survey, and seen at any time throughout the survey period is recorded as ‘present’ (OBS_TYPE=”P”). ‘Instantaneous’ data therefore come from a ‘closed count’ (i.e., representing the density of fishes within a defined area at one point in time). Other data types allow us to integrate data over longer time periods (i.e., to count fishes that are present in or move across the cylinder at some point through the course of the survey). That integrated data allows us to gather systematic data on relative abundance and size distribution of relatively rare, or skittish and/or more mobile species. Depending on the question of interest, we filter the data by its observation type. By default, we pool ‘I’ and ‘N’ data for routine reporting of density estimates, as that allows for the most continuous, comparable dataset, and because we found biomass estimates from I and N data to be relatively similar to those from our previous method (25-m belt transects, which are conducted using an ‘open’ survey method).
2. Data from adjacent SPC cylinder surveys conducted simultaneously are non-independent replicates that are averaged to create a mean estimate for the SPC-pair that is the normal base-level unit of data. In some cases, a site was surveyed by means of two SPC-pairs. However, we still do not consider those independent replicates as those were typically conducted within 20 m or less of each other. When that happens, data are averaged within the SPC-pairs, and then between SPC-pairs to generate site-level estimates.
3. These data are hierarchical in statistical sampling design. Summary statistics (e.g., mean and variances) of survey quantities (e.g., biomass) are calculated from the surveys within each stratum. To pool those up into larger units (e.g., island), we weight each stratum by its relative size (i.e., if a stratum is 50% of the total area for each reporting unit (typically island or atoll) then the weighting factor will be 0.5, and total of all weighting factors sums to 1 ref. 9. Per-strata mean and variance values are aggregated to a higher level (e.g., to island scale) using the formulas below:
4. pooled mean biomass (*X)* across S strata:
  $$X=\sum_{1}^{S}(X_{i}\times w_{i});\;\mathrm{and}$$
5. pooled variance of mean biomass (*VAR)* across S strata:
  $$VAR=\sum_{1}^{S}(VAR_{i}\times w_{i}^{2});$$
  where *X*_*i*_ is the estimate of mean biomass within stratum *i*, *VAR*_*i*_ is the estimated variance of *X*_*i*_ and *w*_*i*_ is the stratum-weighting factor.
6. The SPC is a generalist survey technique, which is the method of choice for Pacific RAMP because our priority indicators are composite groups of reef fishes, as opposed to focusing on individual species. Cryptic, nocturnal, and rare species are not well represented by these surveys.
7. The presence of divers has the potential to alter fish behaviour which can inflate or deflate the counts of fishes, over-counting species that are attracted to divers, as is the case for sharks and jacks in the northwestern Hawaiian Islands, or undercounting species that tend to avoid divers, presumably through a flight response triggered by fishes associating divers with fishing^29,36,37^.
8. The method with which reef substrate complexity is measured has changed over time. To use substrate complexity data from 2010–2017, a linear regression can be applied to generate a standard conversion formula between the two methods^38^.

## Additional Information

**How to cite this article:** Heenan, A. *et al.* Long-term monitoring of coral reef fish assemblages in the Western central pacific. *Sci. Data* 4:170176 doi:10.1038/sdata.2017.176 (2017).

**Publisher’s note:** Springer Nature remains neutral with regard to jurisdictional claims in published maps and institutional affiliations.

## Supplementary Material



Supplementary Information

Supplementary File 1

Supplementary File 2

## Figures and Tables

**Table 1:** Islands and atolls surveyed ordered by U.S. jurisdictional regions and area of hard-bottomed substrate in each of the reef zone strata.

**Region**	**Island**	**Area hard-bottom (<30 m, Ha)**			
		**Backreef**	**Forereef**	**Lagoon**	**Other**
Northwestern Hawaiian Islands	FFS	1,136	16,902	9,728	0
Northwestern Hawaiian Islands	Kure	313	2,438	948	0
Northwestern Hawaiian Islands	Laysan	0	3,400	0	0
Northwestern Hawaiian Islands	Lisianski	0	30,955	0	0
Northwestern Hawaiian Islands	Midway	415	3,294	1,287	0
Northwestern Hawaiian Islands	P&H	1,471	8,498	7,843	0
main Hawaiian Islands	Hawaiʻi	0	16,840	0	0
main Hawaiian Islands	Kahoʻolawe	0	1,200	0	0
main Hawaiian Islands	Kauaʻi	0	18,127	0	0
main Hawaiian Islands	Lānaʻi	0	3,004	0	0
main Hawaiian Islands	Maui	0	11,122	0	0
main Hawaiian Islands	Molokaʻi	0	12,730	0	0
main Hawaiian Islands	Niʻihau	0	9,266	0	0
main Hawaiian Islands	Oʻahu	0	25,119	0	0
Pacific Remote Island Areas	Baker	0	390	0	0
Pacific Remote Island Areas	Howland	0	173	0	0
Pacific Remote Island Areas	Jarvis	0	366	0	0
Pacific Remote Island Areas	Johnston	357	6,574	2,913	0
Pacific Remote Island Areas	Kingman	473	2,298	1,090	13
Pacific Remote Island Areas	Palmyra	1,327	2,793	74	19
Pacific Remote Island Areas	Wake	695	280	307	0
American Samoa	O&O	0	793	0	0
American Samoa	Rose	171	120	104	46
American Samoa	Swains	0	281	0	0
American Samoa	Ta‘ū	0	904	0	0
American Samoa	Tutuila	0	4,182	0	0
Northern Marianas	Agrihan	0	851	0	0
Northern Marianas	AGS	0	744	0	0
Northern Marianas	Asuncion	0	249	0	0
Northern Marianas	FDP	0	138	0	0
Northern Marianas	Maug	0	314	0	0
Northern Marianas	Pagan	0	1,513	0	0
Northern Marianas	Aguijan	0	406	0	0
Southern Marianas	Guam	0	7,296	0	0
Southern Marianas	Rota	0	1,331	0	0
Southern Marianas	Saipan	598	3,539	583	127
Southern Marianas	Tinian	0	1,414	0	0
Other includes substrate types that contribute to the hard-bottom substrate per island that we do not sample, such as channels. The abbreviated islands are FFS=French Frigate Shoals, P&H=Pearl and Hermes, O&O=Ofu and Olosega, AGS=Alamagan, Guguan and Sarigan, FDP=Farallon de Pajaros.					

**Table 2:** Sampling and survey terms and definitions.

**Term**	**Definition**
Survey site data	The mean values of estimated observed quantities from the stationary point counts surveys conducted at each site. Typically derived from a single pair of simultaneous adjacent stationary point counts (i.e., two or more surveys). Sites have associated metadata including depth, visibility, slope, habitat type and complexity and geographic coordinates.
Reporting unit	A collection of survey sites, typically an island or atoll, and in some cases small island groups or sectors of larger islands
Statistical sampling domain	Hard-bottom habitat in <30-m depths
Strata	Reef zone (backreef, forereef, protected slope, lagoon)
	Depth zone (shallow 0–6 m*, mid 6–18 m, deep 18–30 m)
	Sectors (e.g., management units† and stretches of coastline with broadly similar habitat, exposure, and local human population density‡)

*For practical reasons, sites in which the centre point of the survey cylinder is shallower than 1.5 m are not surveyed.

^†^For the island of Guam only.

^‡^Currently only in the main Hawaiian Islands, Tutuila, and Guam. Due to limited replication we typically pool depth zones together for backreef and lagoon zones.

**Table 3:** Number of sites surveyed per island/atolls by reef zone strata (B=backreef, F=forereef, L=lagoon, P=protected slope) per year.

**Region**	**Island**	**2010**				**2011**				**2012**				**2013**				**2014**				**2015**				**2016**				**2017**			
		**B**	**F**	**L**	**PS**	**B**	**F**	**L**	**PS**	**B**	**F**	**L**	**PS**	**B**	**F**	**L**	**PS**	**B**	**F**	**L**	**PS**	**B**	**F**	**L**	**PS**	**B**	**F**	**L**	**PS**	**B**	**F**	**L**	**PS**
Northwestern Hawaiian Islands	FFS		9	8	8		1	3	4		2	3	10					1	16	2	8		6		2		23	6	18				
Northwestern Hawaiian Islands	Kure	3	16	6						3	14	3											8				39						
Northwestern Hawaiian Islands	Laysan						23																8										
Northwestern Hawaiian Islands	Lisianski		25				9				25								28				18				40						
Northwestern Hawaiian Islands	Midway					5	17	8										4	30			2	12										
Northwestern Hawaiian Islands	P&H	7	23	10			9	9		1	15	15										2	21				51	5					
main Hawaiian Islands	Hawaiʻi		43												58								97				59						
main Hawaiian Islands	Kahoʻolawe																										24						
main Hawaiian Islands	Kauaʻi		26												37								20				30						
main Hawaiian Islands	Lāna‘i		16								29				29								15				26						
main Hawaiian Islands	Maui		33								49				34								30				28						
main Hawaiian Islands	Molokaʻi		10								50				39								48				23						
main Hawaiian Islands	Niʻihau		16												26								49				12						
main Hawaiian Islands	Oʻahu		40								35				64								35				54						
Pacific Remote Island Areas	Baker		21								24												36										
Pacific Remote Island Areas	Howland		16								39												34										
Pacific Remote Island Areas	Jarvis		20								42												62				30				28		
Pacific Remote Island Areas	Johnston	4	5	15	15					3	6	13	13										5	7	19								
Pacific Remote Island Areas	Kingman	9	17	7						12	26	11										7	34	8									
Pacific Remote Island Areas	Palmyra		33	2							42												78										
Pacific Remote Island Areas	Wake						30												43												53		
American Samoa	O&O		30								30												52				11						
American Samoa	Rose	6	24	4						15	33											5	37	5			47						
American Samoa	Swains		24								38												32										
American Samoa	Ta‘ū		24								22												46				50						
American Samoa	Tutuila		110								85												157				75						
Northern Marianas	Agrihan						20																								19		
Northern Marianas	Aguijan						13												10												17		
Northern Marianas	Alamagan						5												11												9		
Northern Marianas	Asuncion						20												21												19		
Northern Marianas	FDP						12												11												16		
Southern Marianas	Guam						133												90												66		
Northern Marianas	Guguan						10												11												9		
Northern Marianas	Maug						30												40												38		
Northern Marianas	Pagan						29												43												40		
Southern Marianas	Rota						24												28												28		
Southern Marianas	Saipan						30												45												37		
Southern Marianas	Sarigan						9												11												9		
Southern Marianas	Tinian						19												19												24		
The abbreviated islands are FFS=French Frigate Shoals, P&H=Pearl and Hermes, O&O=Ofu and Olosega and FDP=Farallon de Pajaros.																																	

**Table 4:** Descriptions of data columns in the Pacific RAMP REA fish survey data file

**Data field**	**Descriptor**
REGION	Code for one of the five Pacific Island regions Pacific RAMP surveys. MHI=main Hawaiian Islands, NWHI=northwestern Hawaiian Islands, PRIA=Pacific Remote Island Areas, SAMOA=American Samoa.
ISLAND	Island or atoll surveyed
SITE	The 9-character code for the site surveyed. Combines a 3-letter island code with a site number
LATITUDE	Site latitude in decimal degrees
LONGITUDE	Site longitude in decimal degrees
REEF_ZONE	The reef zone for the given site (forereef, backreef, lagoon, protected slope)
DEPTH_BIN	Classification of depth (shallow [0–6 m], mid [6–18 m], deep [18–30 m]) based on midpoint between minimum and maximum depths of replicates at this site
SITEVISITID	Unique numeric identifier for each site visit record. A site visit represents a single survey at a particular site
DATE	Date the survey was conducted
OBS_YEAR	Year the survey was conducted
DIVER	Unique numeric code for diver conducting survey
REPLICATEID	Unique identifier for the point count sample within a fish survey
REP	Letter associated to a pair of adjacent point counts (mostly one pair per survey, thus ‘A’; can also be ‘B’ in cases where a survey involved more than one SPC-pair)
DEPTH_M	Depth of survey area in meters
HARD_CORAL	Percentage of hard coral that is part of the overall benthic cover
MA	Percentage of macroalgae that is part of the overall benthic cover
CCA	Percentage of crustose coralline algae that is part of the overall benthic cover
SAND	Percentage of sand algae that is part of the overall benthic cover
OTHER	Percentage of other categories (turf algae, soft coral and cyanobacteria) that are part of the overall benthic cover
HABITAT_CODE	Habitat Type. AGR (aggregate reef), APR (aggregate patch reef), APS (aggregate patch reefs), MIX (mixed habitat), PAV (pavement), PPR (pavement with patch reefs), PSC (pavement with sand channels), ROB (rock boulder), RRB (reef rubble), SAG (spur and groove), SCR (scattered coral/rock), UNK (unknown) WAL (Wall). This represents the general area in which the survey is conducted, rather than the exact area of the survey cylinders. Nominally, this is based on thinking of the survey itself as being at the centre of a 50 m*50 m cell and habitat code is for the entire cell.
CURRENT_STRENGTH	Strength of water current as qualitatively assessed by diver (None, Slight, Moderate, High). Contains missing values prior to 2013.
VISIBILITY_M	Visibility measured in meters. Between 2010 and 2014, this was estimated by divers. From 2015 onwards, horizontal visibility has been measured using a Secchi disc. All estimates capped at 30 m.
MIN_DEPTH_M	Minimum slope depth in meters
MAX_DEPTH_M	Maximum slope depth in meters
COMPLEXITY	Visual estimate of complexity on a six point scale (1:6) from surveys in 2010 and 2011
SUBSTRATE_HEIGHT_0	Visual estimate of percentage of survey cylinder between 0 and 20 cm in relief from surveys 2012 onwards
SUBSTRATE_HEIGHT_20	Visual estimate of percentage of survey cylinder between 20 and 50 cm in relief from surveys 2012 onwards
SUBSTRATE_HEIGHT_50	Visual estimate of percentage of survey cylinder between 50 and 100 cm in relief from surveys 2012 onwards
SUBSTRATE_HEIGHT_100	Visual estimate of percentage of survey cylinder between 100 and 150 cm in relief from surveys 2012 onwards
SUBSTRATE_HEIGHT_150	Visual estimate of percentage of survey cylinder over 150 cm in relief from surveys 2012 onwards
MAX_HEIGHT	Highest elevation point in SPC cylinder measured in centimeters from surveys 2012 onwards
URCHIN_DACOR	Semi-quantitative estimate of free urchin abundance (D: Dominant [>100], A: Abundant [51–100], C: Common [21–50], O: Occasional [6–20], R: Rare [<5]) from surveys 2012 onwards
BORING_URCHIN_DACOR	Semi-quantitative estimate of boring urchin abundance (D: Dominant [>500], A: Abundant [251–500], C: Common [101–250], O: Occasional [26–100], R: Rare [<25]) from surveys 2012 onwards
SPECIES	4-letter species code for data entry
TAXONNAME	Scientific name at time the species was added to the database
COMMON_FAMILY	Common name of family
FAMILY	Taxonomic family classification
CONSUMER_GROUP	Consumer groupings used for Pacific RAMP reporting (Primary Consumer, Secondary Consumer, Planktivore, Piscivore)
LW_A	Parameter ‘a’ used in length-weight calculations
LW_B	Parameter ‘b’ used in length-weight calculations
LMAX	Maximum length of the fish derived largely from FishBase
LENGTH_CONVERSION_FACTOR	Factor to convert total length to correct form (standard length, fork length) if the length weight A and B parameters to convert length to biomass are not in total length
COUNT	Number of fish observed of this size and species
SIZE_TL_CM	Estimated total length, for fishes this is from the tip of the snout to the tip of the longer lobe of the caudal fin, reported in centimeters. Length for rays (e.g., Myliobatidae, Dasyatidae) is measured from pectoral fin tip to pectoral fin tip
OBS_TYPE	A single letter representation of the observation type (I: Instantaneous, N: Non-Instantaneous, F: species first entering the cylinder 5–10 min after start of survey, T: species entering cylinder 10–30 min after start of surveys, P: Present in vicinity of survey
