# Supplementary material for: Long-term monitoring of coral reef fish assemblages in the Western central pacific
Source: Sci Data. 2017 Dec 5;4:170176. doi: 10.1038/sdata.2017.176 (PMC5716063; doi:10.1038/sdata.2017.176)
Supplement: Supplementary Information [file sdata2017176-s2.docx]

Supplementary File 3.

The majority of fish survey data collected for RAMP before 2010 was from belt transect surveys collected under a haphazardly fixed survey design. The decision to change to the SPC method was based on a comparison of the abundance and biomass estimates derived from both methods which were simultaneously conducted at the same sites. Between 2007-2009, 332 such paired SPC and belt comparison surveys were made. A summary of the biomass and density estimates for each method and the ratio between the two is in Table S3.1. The quality of data collected by both methods was compared in the following manner, as variability within the location/strata combination relative to the mean estimate, the goodness of fit for the models of biomass and abundance, percentage of zeros (the proportion of surveys where no individuals of individual taxa were recorded and as relative precision (SPC co-efficient of variation / Belt co-efficient of variation) – relative precision estimates of > 1 indicate that the SPC is more variable and < 1 mean the belt estimates are more variable. To compare the estimates of biomass and abundance of survey target groups, linear models were fitted to with Archipelago, Island(Archipelago) and Depth strata as explanatory factors. For all fish biomass, functional groups based on diet and other fisheries target groups of interest, models were fit to estimates of biomass. For smaller, largely non-target group, abundance was used. Prior to model fitting all data were ln(x+1) transformed.

Table S3.1 **A comparison of the biomass (gm-2) and abundance (per 100m2) estimates from paired SPC and belt surveys**. The root mean square error (RMSE) was used as the measure variability in these data when variance due to model factors was removed, i.e.is equivalent to residual standard deviation among sites within an island and within a depth strata.. ‘CV’ in the table below is coefficient of variation (=RMSE/Mean for untransformed data). CVs for transformed data were calculated after back-transforming [exp(x)-1] mean and error. As back-transformed error is not symmetrical around the mean, the average of upper and lower error range was used in place of RMSE in calculation of CV. ‘Relative precision’ of BLT and SPC were determined by dividing (SPC CV) by (BLT CV). Values >1 means SPC data is more variable, <1 means BLT data is more variable. Model r^2^ is the proportion of total error that can be attributed to model factors (archipelago, depth, island[archipelago]). High r^2^ is equivalent to remaining error (1-r^2^) being low. As remaining error comes from the process of sampling rather than from real differences among sites, it is better if that is low (i.e. r^2^ is high). Low CV together with high r^2^ - i.e. relatively low variability, but good ability to distinguish among locations - is therefore a strong indication of good data quality.

|  |  | Untransformed data | | |  | |  | Transformed data Ln(x+1) | | | | |
| --- | --- | --- | --- | --- | --- | --- | --- | --- | --- | --- | --- | --- |
|  |  | Mean (CV) | | %ZEROS | |  | | r2-adj |  | CV |  |  |
|  | **Taxa/Grouping** | **Belt** | **SPC** | **Belt** | | **SPC** | | **Belt** | **SPC** | **Belt** | **SPC** | **Relative Precision** |
| Biomass | All fish | 130.6 (118%) | 130.6 (95%) | 0 | | 0 | | 0.37 | 0.43 | 0.86 | 0.84 | 0.98 |
|  | Herbivorous fish | 43.6 (123%) | 36.1 (76%) | 0 | | 0 | | 0.16 | 0.25 | 0.95 | 0.88 | 0.92 |
|  | Planktivore | 25.6 (320%) | 25.5 (209%) | 0.02 | | 0.02 | | 0.45 | 0.44 | 1.32 | 1.42 | 1.08 |
|  | Piscivore | 36.8 (224%) | 50.3 (162%) | 0.07 | | 0.03 | | 0.37 | 0.55 | 1.65 | 1.34 | 0.81 |
|  | Secondary Consum | 24.6 (308%) | 18.5 (178%) | 0 | | 0 | | 0.19 | 0.17 | 1.05 | 0.89 | 0.85 |
|  | Grouper | 5.6 (107%) | 7.8 (104%) | 0.27 | | 0.21 | | 0.55 | 0.65 | 1.13 | 0.95 | 0.84 |
|  | Jack (not Zooplankti) | 6.3 (403%) | 7.9 (278%) | 0.76 | | 0.70 | | 0.25 | 0.35 | 2.78 | 2.45 | 0.88 |
|  | Emperors | 1.6 (539%) | 1.7 (414%) | 0.81 | | 0.76 | | 0.05 | 0.10 | 3.17 | 2.66 | 0.84 |
|  | Goatfish | 1.7 (234%) | 1.9 (263%) | 0.41 | | 0.29 | | 0.16 | 0.20 | 1.77 | 1.66 | 0.94 |
|  | Parrot | 15.2 (120%) | 13.4 (114%) | 0.14 | | 0.12 | | 0.15 | 0.16 | 1.66 | 1.53 | 0.92 |
|  | Surgeon | 23.0 (197%) | 18.4 (105%) | 0.01 | | 0.01 | | 0.27 | 0.36 | 0.98 | 0.92 | 0.94 |
|  | Snapper | 11.9 (195%) | 15.5 (165%) | 0.42 | | 0.34 | | 0.35 | 0.47 | 2.03 | 1.75 | 0.86 |
|  | Soldier/Squirrel | 6.3 (330%) | 5.8 (234%) | 0.55 | | 0.52 | | 0.33 | 0.36 | 2.04 | 1.96 | 0.96 |
| Abund | Angelfish | 5.6 (122%) | 5.7 (102%) | 0.24 | | 0.14 | | 0.36 | 0.36 | 1.15 | 0.95 | 0.83 |
|  | Butterflyfish | 3.9 (121%) | 3.4 (95%) | 0.26 | | 0.11 | | 0.15 | 0.18 | 1.16 | 0.93 | 0.80 |
|  | Planktiv. Damsel | 189.9 (103%) | 133.9 (94%) | 0.05 | | 0.05 | | 0.48 | 0.46 | 0.88 | 0.85 | 0.97 |
|  | Benthic Damsel | 24.4 (116%) | 20.9 (128%) | 0.08 | | 0.10 | | 0.22 | 0.24 | 1.03 | 1.14 | 1.11 |
|  | Hawkfish | 6.2 (106%) | 4.7 (143%) | 0.26 | | 0.26 | | 0.52 | 0.43 | 1.01 | 1.29 | 1.28 |
|  | Wrasse | 46.5 (106%) | 28.8 (140%) | 0 | | 0 | | 0.36 | 0.23 | 0.72 | 0.86 | 1.24 |
